# Harnessing Metabolomics to Describe the Pathophysiology Underlying Progression in Diabetic Kidney Disease

**DOI:** 10.1007/s11892-021-01390-8

**Published:** 2021-05-11

**Authors:** Sho Hasegawa, Reiko Inagi

**Affiliations:** 1 Division of Chronic Kidney Disease Pathophysiology, The University of Tokyo Graduate School of Medicine, 7-3-1 Hongo, Bunkyo-ku, Tokyo, 113-8655 Japan; 2 Division of Nephrology and Endocrinology, The University of Tokyo Graduate School of Medicine, Tokyo, Japan

**Keywords:** Diabetic kidney disease, Metabolomics, Mitochondria, Organelle crosstalk, Uremic toxin, Biomarker

## Abstract

**Purpose of Review:**

Diabetic kidney disease (DKD), a leading cause of end-stage kidney disease, is the result of metabolic network alterations in the kidney. Therefore, metabolomics is an effective tool for understanding its pathophysiology, finding key biomarkers, and developing a new treatment strategy. In this review, we summarize the application of metabolomics to DKD research.

**Recent Findings:**

Alterations in renal energy metabolism including the accumulation of tricarboxylic acid cycle and glucose metabolites are observed in the early stage of DKD, and they finally lead to mitochondrial dysfunction in advanced DKD. Mitochondrial fission-fusion imbalance and dysregulated organelle crosstalk might contribute to this process. Moreover, metabolomics has identified several uremic toxins including phenyl sulfate and tryptophan derivatives as promising biomarkers that mediate DKD progression.

**Summary:**

Recent advances in metabolomics have clarified the role of dysregulated energy metabolism and uremic toxins in DKD pathophysiology. Integration of multi-omics data will provide additional information for identifying critical drivers of DKD.

## Introduction

Diabetic kidney disease (DKD) is a complication of diabetes mellitus and one of the leading causes of end-stage kidney disease (ESKD) [1]. As the pathophysiology of DKD is complex, multifactorial, and heterogeneous, it is difficult to develop effective therapeutic strategies. In recent years, sodium-glucose cotransporter 2 (SGLT2) inhibitors showed a strong protection against DKD progression in several clinical studies [2–6]. SGLT2 inhibitors reduce energy consumption in proximal tubules by preventing the reabsorption of filtered glucose, suggesting dysregulated energy metabolism in the kidney might play a direct role in DKD progression.

The metabolome is the complete set of small molecules found within a biological specimen, and it reflects the phenotype of biological activities [7]. The systematic analysis of metabolites is called metabolomics. DKD is caused by metabolic network alterations in the kidney, making metabolomics an effective tool for understanding its pathophysiology, finding key biomarkers, and developing a new treatment strategy [8]. In this review, we summarize the application of metabolomics with a focus on the role of dysregulated energy metabolism and uremic toxins in DKD progression.

## Dysregulated Energy Metabolism and Mitochondrial Dysfunction in DKD

**Fig. 1.**
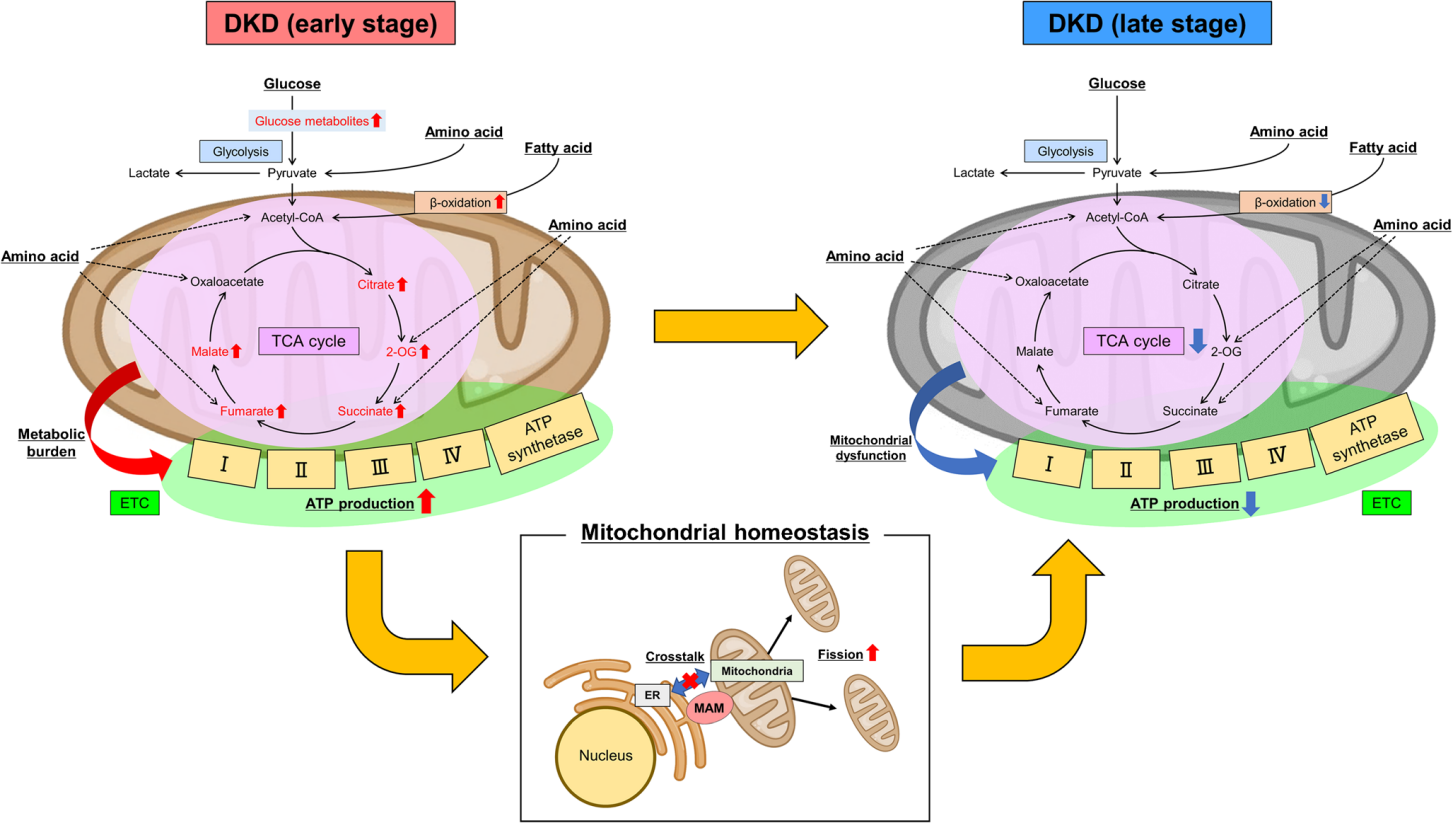
Dysregulated energy metabolism and mitochondrial dysfunction in diabetic kidney disease (DKD). In the early stage of DKD, TCA cycle and glucose metabolites are accumulated in renal tissues. The metabolic burden induces the imbalance of mitochondrial fission-fusion and dysregulated crosstalk between ER and mitochondria through MAMs, which finally leads to mitochondrial dysfunction in the late stage of DKD. TCA cycle, tricarboxylic acid cycle; ETC, the electron transport chain; ER, endoplasmic reticulum; MAM, mitochondria-associated membrane

Mitochondria are the centers of energy production. Energy substrates including glucose, amino acids, and fatty acids enter the tricarboxylic acid (TCA) cycle (Fig. 1). The TCA cycle supplies the electron transport chain with the reduced forms of nicotinamide adenine dinucleotide (NADH) and flavin adenine dinucleotide (FADH2). The electron transport chain, a series of electron transporters (complex I–IV) in the inner mitochondrial membrane, shuttles electrons from NADH and FADH2 to molecular oxygen. The proton gradient between the mitochondrial matrix and intermembrane space generated during this process is used by adenosine triphosphate (ATP) synthetase to produce energy. This process is called mitochondrial respiration or oxidative phosphorylation (OXPHOS).

Mitochondria are abundant in the proximal tubular cells of kidneys due to the high amount of energy required for reabsorption of glucose and sodium. Since metabolic alterations in renal tissues induced by hyperglycemia and dyslipidemia play critical roles in DKD progression, metabolomics is an efficient strategy for a comprehensive understanding of the mitochondria and energy metabolism dynamics in the kidney [7].

Sharma et al. analyzed the urine metabolome of advanced DKD patients and found that 13 metabolites were significantly lower in DKD patients than in healthy controls [9]. Majority of the 13 metabolites were localized or transported into the mitochondria, suggesting that mitochondrial dysfunction was associated with DKD progression. In fact, the expression of peroxisome proliferator-activated receptor gamma coactivator 1α (PGC1α) and cytochrome c was reduced in the renal tissues of DKD patients. Moreover, there was less mitochondrial DNA in urine exosomes obtained from DKD patients than those obtained from healthy controls [9].

Mitochondrial dysfunction is a common characteristic in the development of chronic kidney disease (CKD) (Fig. 1). Transcriptome analysis of human kidney samples from CKD patients revealed that defective fatty acid oxidation (FAO) played a key role in kidney fibrosis development [10]. Recently, single-cell RNA sequencing of mouse kidney fibrotic models clarified that impaired energy metabolism (FAO and OXPHOS) coupled with poor cell differentiation of proximal tubules is a critical driver of kidney fibrosis [11••].

In contrast, incipient DKD is different from advanced DKD in terms of renal energy metabolism (Fig. 1). Li et al. analyzed urine metabolites at 6, 8, 10, 12, and 16 weeks in db/db and control mice [12]. The urinary concentrations of TCA cycle intermediates such as citrate and succinate were lower in the db/db mice than in the controls at the end of week 16. However, these TCA cycle intermediates showed gradual elevation from week 6 to week 12, suggesting that the TCA cycle was active in the early stage of DKD. Moreover, metabolic flux analysis with isotope-labeled glucose, pyruvate, and fatty acid (palmitate) revealed that TCA cycle, glycolysis, and FAO fluxes in the renal tissues were higher in db/db mice than in control mice [13].

The remained question is when and how the increased renal metabolic flux in the early stage of diabetes finally leads to mitochondrial dysfunction observed in advanced DKD. Several studies have suggested that the accumulation of TCA cycle and glycolysis intermediates is toxic and directly causes kidney injury. For example, succinate, a TCA cycle intermediate, is a ligand of the G protein-coupled receptor GPR 91 and contributes to blood pressure increase [14–16] and NADPH oxidase 4 (Nox4)-induced mitochondrial damage [17, 18] through the activation of renin-angiotensin-aldosterone systems. Moreover, fumarate, another TCA cycle metabolite, has been shown to accumulate in the renal tissues of F1 Akita mice partly due to the Nox4-induced downregulation of fumarate hydroxylase expression, which stimulates endoplasmic reticulum (ER) stress, matrix gene expressions, and profibrotic signaling in the kidney [19]. Furthermore, the accumulation of the glucose metabolites sorbitol, methylglyoxal, and diacylglycerol might induce kidney disease progression. According to the proteomics data on glomeruli from patients with diabetes, glycolysis-related enzymes were higher in individuals with prolonged diabetes but no kidney disease than in patients with DKD [20]. In particular, the expression and activity of pyruvate kinase M2 (PKM2), which catalyzes the last step of glycolysis, were upregulated in these patients. In the animal studies, podocyte-specific Pkm2-knockout mice with diabetes developed severe glomerular injuries. Furthermore, pharmacological activation of Pkm2 reversed hyperglycemia-induced elevation in glucose metabolites along with the prevention of mitochondrial dysfunction and glomerular injuries.

Thus, the accumulation of TCA cycle and glucose metabolites in renal tissues could be a therapeutic target in the early stage of DKD. This hypothesis was further confirmed by the metabolome analysis of BTBR ob/ob mice by our group in which we found that SGLT2 inhibitors reversed the accumulation of TCA cycle metabolites and reduced oxidative stress in renal tissues [21]. This metabolic effect occurs mainly because SGLT2 inhibitors decrease the energy demand in proximal tubules by reducing the reabsorption of glucose and sodium.

The potential benefits of reducing TCA cycle flux during the early stage of DKD observed in animal studies support the hypothesis that metabolic reprogramming by hypoxia-inducible factor-prolyl hydroxylase (HIF-PH) inhibitors, novel therapeutic agents for renal anemia [22], may attenuate the harmful effects of metabolic burden in patients with incipient DKD. In the hypoxic environment, HIF is activated and induces metabolic reprogramming from the TCA cycle to glycolysis to reduce oxygen consumption of each cell. As HIF-PH inhibitors induce HIF activation in the normoxic environment, metabolic reprogramming might reverse the accumulation of TCA cycle metabolites. In our animal studies, transcriptome and metabolome analysis of renal tissues revealed that HIF-PH inhibitors counteract the renal metabolism alterations occurring in the early stages of DKD. The accumulation of TCA cycle and glucose metabolites was prevented by the administration of HIF-PH inhibitors. Additionally, this metabolic change was associated with an improvement in renal pathological abnormalities such as glomerular hypertrophy and basement membrane thickening [23••]. Our results cannot be confirmed in clinical settings because HIF-PH inhibitors are therapeutic agents used for renal anemia and administered only in anemic patients with late-stage DKD. However, our study suggests that metabolic reprogramming toward reducing TCA cycle and glucose metabolites accumulation may serve as a potential intervention that targets the dysregulated renal energy metabolism in the early stages of DKD.

Although further studies are needed to clarify the precise mechanism of how the dysregulated energy metabolism in incipient DKD leads to mitochondrial dysfunction in advanced DKD, one possible mechanism is the imbalance between mitochondrial fission and fusion in the diabetic state (Fig. 1). Mitochondria continuously change their morphology by repeated fission and fusion that are regulated by various molecules such as dynamin-related protein 1 (Drp1), mitochondrial fission 1 protein (Fis1), mitofusin 1 (Mfn1), mitofusin 2 (Mfn2), and OPA1 mitochondrial dynamin-like GTPase (OPA1). The increase in mitochondrial fission is observed in the proximal tubules of DKD, in association with membrane potential decrease, reduced ATP production, and cellular apoptosis [24]. Wang et al. showed that mitochondrial fission is induced by Drp1 recruitment to the mitochondria in the podocytes of DKD, which is partly mediated by Rho-associated coiled coil-containing protein kinase 1 (ROCK1). Deletion of ROCK1 in podocytes suppressed mitochondrial fission and progression of DKD [25]. However, mitochondrial fission may not only be an aggravating factor but also an adaptive response to metabolic stress induced by diabetes. Wang et al. revealed that deletion of Drp1 in the liver protects mice from diet-induced obesity and metabolic deterioration [26]. Thus, the mitochondrial fission is considered a compensatory response against metabolic stress, and mitochondrial dysfunction would occur when the compensation reaches a limit. There is a possibility that metabolic reprogramming toward reducing TCA cycle and glucose metabolism in the diabetic state might reduce the mitochondrial burden and relieve the need for adaptive responses including mitochondrial fission.

Interestingly, the mitochondrial fusion protein Mfn2 is deeply involved in the formation of organelle contact sites between the ER and mitochondria [27] called mitochondria-associated membranes (MAMs) (Fig. 1). Recent studies have clarified that MAMs maintain cellular homeostasis by regulating lipid transport and calcium signaling transduction between the ER and mitochondria [28]. Thus, the organelle crosstalk between the ER and mitochondria might have important roles in mitochondrial homeostasis including fission-fusion balance. Unraveling the interaction among metabolic stress, mitochondrial homeostasis, and organelle crosstalk would provide important insights into how the dysregulated energy metabolism in incipient DKD leads to mitochondrial dysfunction in advanced DKD.

## Uremic Toxin as Biomarkers and Pathological Factors in DKD

The urinary excretion of waste products is gradually reduced during CKD progression. The waste products having bad effects on human’s bodies are called uremic toxins. As most uremic toxins are metabolites produced from dietary contents by the gut microbiota [29], metabolomics is a strong strategy to understand the changes in the uremic toxin levels in the course of kidney diseases including DKD. Niewczas et al. analyzed plasma metabolomic profiles of patients with type 2 diabetes and CKD. Some plasma metabolites including p-Cresol sulfate (an amino-acid derivative), pseudouridine (a nucleotide derivative), and myo-inositol (a carbohydrate derivative) were identified as uremic solutes associated with the progression to ESKD after adjustment for renal functions and blood glucose control [30]. Additionally, they analyzed serum metabolomic profiles of patients with type 1 diabetes and CKD. Serum levels of 7 metabolites including tryptophan and tyrosine derivatives were associated with renal function decline and time to ESKD, independent of the relevant clinical covariates [31]. Although it is difficult to judge whether increased uremic toxins are the cause or result, a recent study suggests that phenyl sulfate, one of the tyrosine derivatives, is a predictive marker and also mediates DKD progression [32••]. Phenyl sulfate levels showed a significant correlation with 2-year progression of albuminuria in diabetic patients with microalbuminuria. Furthermore, inhibition of tyrosine phenol-lyase, a bacterial enzyme responsible for producing phenol from tyrosine, reduced urinary albumin levels in animal studies, suggesting that gut microbiota could be a therapeutic target.

Tryptophan derivatives are considered another promising therapeutic target against DKD progression. The serum level of kynurenine produced from tryptophan was associated with overt proteinuria in DKD [33]. As the kidney function declines in patients with type 2 diabetes, increase in serum kynurenine levels and decrease in serum tryptophan levels were observed [34]. Korstanje et al. showed that the glomerular expression of kynurenine 3-mono-oxygenase (KMO) was reduced in patients with diabetes [35]. KMO is the enzyme that catalyzes the hydroxylation of kynurenine. Thus, loss of KMO leads to an increase in kynurenine and kynurenic acid. As KMO knockout mice showed foot process effacement and proteinuria, the kynurenine pathway might have an important role in kidney disease progression of patients with diabetes.

In the future, network analysis using multi-omics data from patients will be important. Saito et al. identified mouse double minute 2 homolog (MDM2) as a key factor in the DKD pathophysiology by integrating their urinary metabolome data with publicly available human protein-protein interaction database [36]. In fact, the expression of *MDM2* gene was significantly reduced in the renal tissues of DKD patients compared with controls. Moreover, podocyte-specific and tubule-specific *Mdm2*-knockout mice showed severe glomerular and tubular dysfunction, respectively. Thus, integrating omics information might be a good strategy for identifying critical drivers of kidney diseases. Ideally, multi-omics data from the same patient cohort should be collected for a comprehensive understanding of DKD pathophysiology.

## Conclusions

Recent advances in metabolomics have clarified the role of dysregulated energy metabolism and uremic toxins in DKD pathophysiology. Of note, the energy metabolic state of the kidney is different between early- and late-stage DKD. Unraveling the interaction among metabolic stress, mitochondrial homeostasis, and organelle crosstalk in the kidney is necessary for further understanding of the energy metabolic dynamics during DKD progression. Although metabolomics has identified several uremic toxins including phenyl sulfate and tryptophan derivatives as promising biomarkers that mediate DKD progression, network analysis using multi-omics data will provide additional information for finding critical drivers of kidney diseases.

## References

[1] Nichols GA, Deruaz-Luyet A, Hauske SJ, Brodovicz KG (2018). The association between estimated glomerular filtration rate, albuminuria, and risk of cardiovascular hospitalizations and all-cause mortality among patients with type 2 diabetes. J Diabetes Complicat.

[2] Wanner C, Inzucchi SE, Lachin JM, Fitchett D, von Eynatten M, Mattheus M, Johansen OE, Woerle HJ, Broedl UC, Zinman B (2016). Empagliflozin and progression of kidney disease in type 2 diabetes. N Engl J Med.

[3] Neal B, Perkovic V, Mahaffey KW, de Zeeuw D, Fulcher G, Erondu N, Shaw W, Law G, Desai M, Matthews DR (2017). Canagliflozin and cardiovascular and renal events in type 2 diabetes. N Engl J Med.

[4] Perkovic V, Jardine MJ, Neal B, Bompoint S, Heerspink HJL, Charytan DM, Edwards R, Agarwal R, Bakris G, Bull S, Cannon CP, Capuano G, Chu PL, de Zeeuw D, Greene T, Levin A, Pollock C, Wheeler DC, Yavin Y, Zhang H, Zinman B, Meininger G, Brenner BM, Mahaffey KW (2019). Canagliflozin and renal outcomes in type 2 diabetes and nephropathy. N Engl J Med.

[5] Wiviott SD, Raz I, Bonaca MP, Mosenzon O, Kato ET, Cahn A, Silverman MG, Zelniker TA, Kuder JF, Murphy SA, Bhatt DL, Leiter LA, McGuire DK, Wilding JPH, Ruff CT, Gause-Nilsson IAM, Fredriksson M, Johansson PA, Langkilde AM, Sabatine MS (2019). Dapagliflozin and cardiovascular outcomes in type 2 diabetes. N Engl J Med.

[6] Heerspink HJL, Stefansson BV, Correa-Rotter R, Chertow GM, Greene T, Hou FF (2020). Dapagliflozin in patients with chronic kidney disease. N Engl J Med.

[7] Kalim S, Rhee EP (2017). An overview of renal metabolomics. Kidney Int.

[8] Darshi M, Van Espen B, Sharma K (2016). Metabolomics in diabetic kidney disease: unraveling the biochemistry of a silent killer. Am J Nephrol.

[9] Sharma K, Karl B, Mathew AV, Gangoiti JA, Wassel CL, Saito R, Pu M, Sharma S, You YH, Wang L, Diamond-Stanic M, Lindenmeyer MT, Forsblom C, Wu W, Ix JH, Ideker T, Kopp JB, Nigam SK, Cohen CD, Groop PH, Barshop BA, Natarajan L, Nyhan WL, Naviaux RK (2013). Metabolomics reveals signature of mitochondrial dysfunction in diabetic kidney disease. J Am Soc Nephrol.

[10] Kang HM, Ahn SH, Choi P, Ko YA, Han SH, Chinga F, Park ASD, Tao J, Sharma K, Pullman J, Bottinger EP, Goldberg IJ, Susztak K (2015). Defective fatty acid oxidation in renal tubular epithelial cells has a key role in kidney fibrosis development. Nat Med.

[11.••] Dhillon P, Park J, Hurtado Del Pozo C, Li L, Doke T, Huang S (2021). The nuclear receptor ESRRA protects from kidney disease by coupling metabolism and differentiation. Cell Metab.

[12] Li M, Wang X, Aa J, Qin W, Zha W, Ge Y, Liu L, Zheng T, Cao B, Shi J, Zhao C, Wang X, Yu X, Wang G, Liu Z (2013). GC/TOFMS analysis of metabolites in serum and urine reveals metabolic perturbation of TCA cycle in db/db mice involved in diabetic nephropathy. Am J Physiol Ren Physiol.

[13] Sas KM, Kayampilly P, Byun J, Nair V, Hinder LM, Hur J, Zhang H, Lin C, Qi NR, Michailidis G, Groop PH, Nelson RG, Darshi M, Sharma K, Schelling JR, Sedor JR, Pop-Busui R, Weinberg JM, Soleimanpour SA, Abcouwer SF, Gardner TW, Burant CF, Feldman EL, Kretzler M, Brosius FC, Pennathur S (2016). Tissue-specific metabolic reprogramming drives nutrient flux in diabetic complications. JCI Insight.

[14] He W, Miao FJ, Lin DC, Schwandner RT, Wang Z, Gao J (2004). Citric acid cycle intermediates as ligands for orphan G-protein-coupled receptors. Nature..

[15] Toma I, Kang JJ, Sipos A, Vargas S, Bansal E, Hanner F, Meer E, Peti-Peterdi J (2008). Succinate receptor GPR91 provides a direct link between high glucose levels and renin release in murine and rabbit kidney. J Clin Invest.

[16] Peti-Peterdi J (2010). High glucose and renin release: the role of succinate and GPR91. Kidney Int.

[17] de Cavanagh EM, Inserra F, Ferder M, Ferder L (2007). From mitochondria to disease: role of the renin-angiotensin system. Am J Nephrol.

[18] Lee DY, Wauquier F, Eid AA, Roman LJ, Ghosh-Choudhury G, Khazim K, Block K, Gorin Y (2013). Nox4 NADPH oxidase mediates peroxynitrite-dependent uncoupling of endothelial nitric-oxide synthase and fibronectin expression in response to angiotensin II: role of mitochondrial reactive oxygen species. J Biol Chem.

[19] You YH, Quach T, Saito R, Pham J, Sharma K (2016). Metabolomics reveals a key role for fumarate in mediating the effects of NADPH oxidase 4 in diabetic kidney disease. J Am Soc Nephrol.

[20] Qi W, Keenan HA, Li Q, Ishikado A, Kannt A, Sadowski T, Yorek MA, Wu IH, Lockhart S, Coppey LJ, Pfenninger A, Liew CW, Qiang G, Burkart AM, Hastings S, Pober D, Cahill C, Niewczas MA, Israelsen WJ, Tinsley L, Stillman IE, Amenta PS, Feener EP, Vander Heiden MG, Stanton RC, King GL (2017). Pyruvate kinase M2 activation may protect against the progression of diabetic glomerular pathology and mitochondrial dysfunction. Nat Med.

[21] Tanaka S, Sugiura Y, Saito H, Sugahara M, Higashijima Y, Yamaguchi J, Inagi R, Suematsu M, Nangaku M, Tanaka T (2018). Sodium-glucose cotransporter 2 inhibition normalizes glucose metabolism and suppresses oxidative stress in the kidneys of diabetic mice. Kidney Int.

[22] Hasegawa S, Tanaka T, Nangaku M (2018). Hypoxia-inducible factor stabilizers for treating anemia of chronic kidney disease. Curr Opin Nephrol Hypertens.

[23.••] Hasegawa S, Tanaka T, Saito T, Fukui K, Wakashima T, Susaki EA (2020). The oral hypoxia-inducible factor prolyl hydroxylase inhibitor enarodustat counteracts alterations in renal energy metabolism in the early stages of diabetic kidney disease. Kidney Int.

[24] Coughlan MT, Nguyen TV, Penfold SA, Higgins GC, Thallas-Bonke V, Tan SM, van Bergen NJ, Sourris KC, Harcourt BE, Thorburn DR, Trounce IA, Cooper ME, Forbes JM (2016). Mapping time-course mitochondrial adaptations in the kidney in experimental diabetes. Clin Sci (Lond).

[25] Wang W, Wang Y, Long J, Wang J, Haudek SB, Overbeek P, Chang BHJ, Schumacker PT, Danesh FR (2012). Mitochondrial fission triggered by hyperglycemia is mediated by ROCK1 activation in podocytes and endothelial cells. Cell Metab.

[26] Wang L, Ishihara T, Ibayashi Y, Tatsushima K, Setoyama D, Hanada Y, Takeichi Y, Sakamoto S, Yokota S, Mihara K, Kang D, Ishihara N, Takayanagi R, Nomura M (2015). Disruption of mitochondrial fission in the liver protects mice from diet-induced obesity and metabolic deterioration. Diabetologia..

[27] de Brito OM, Scorrano L (2008). Mitofusin 2 tethers endoplasmic reticulum to mitochondria. Nature..

[28] Hasegawa S, Inagi R (2020). Organelle stress and crosstalk in kidney disease. Kidney360.

[29] Hasegawa S, Jao TM, Inagi R (2017). Dietary metabolites and chronic kidney disease. Nutrients..

[30] Niewczas MA, Sirich TL, Mathew AV, Skupien J, Mohney RP, Warram JH, Smiles A, Huang X, Walker W, Byun J, Karoly ED, Kensicki EM, Berry GT, Bonventre JV, Pennathur S, Meyer TW, Krolewski AS (2014). Uremic solutes and risk of end-stage renal disease in type 2 diabetes: metabolomic study. Kidney Int.

[31] Niewczas MA, Mathew AV, Croall S, Byun J, Major M, Sabisetti VS, Smiles A, Bonventre JV, Pennathur S, Krolewski AS (2017). Circulating modified metabolites and a risk of ESRD in patients with type 1 diabetes and chronic kidney disease. Diabetes Care.

[32.••] Kikuchi K, Saigusa D, Kanemitsu Y, Matsumoto Y, Thanai P, Suzuki N (2019). Gut microbiome-derived phenyl sulfate contributes to albuminuria in diabetic kidney disease. Nat Commun.

[33] Hirayama A, Nakashima E, Sugimoto M, Akiyama S, Sato W, Maruyama S, Matsuo S, Tomita M, Yuzawa Y, Soga T (2012). Metabolic profiling reveals new serum biomarkers for differentiating diabetic nephropathy. Anal Bioanal Chem.

[34] Debnath S, Velagapudi C, Redus L, Thameem F, Kasinath B, Hura CE, Lorenzo C, Abboud HE, O’Connor JC (2017). Tryptophan metabolism in patients with chronic kidney disease secondary to type 2 diabetes: relationship to inflammatory markers. Int J Tryptophan Res.

[35] Korstanje R, Deutsch K, Bolanos-Palmieri P, Hanke N, Schroder P, Staggs L, Bräsen JH, Roberts ISD, Sheehan S, Savage H, Haller H, Schiffer M (2016). Loss of kynurenine 3-mono-oxygenase causes proteinuria. J Am Soc Nephrol.

[36] Saito R, Rocanin-Arjo A, You YH, Darshi M, Van Espen B, Miyamoto S (2016). Systems biology analysis reveals role of MDM2 in diabetic nephropathy. JCI Insight.

